# Respiratory Fluoroquinolones Monotherapy vs. β-Lactams With or Without Macrolides for Hospitalized Community-Acquired Pneumonia Patients: A Meta-Analysis

**DOI:** 10.3389/fphar.2019.00489

**Published:** 2019-05-08

**Authors:** Sitong Liu, Xiang Tong, Yao Ma, Dongguang Wang, Jizhen Huang, Li Zhang, Man Wu, Lei Wang, Tao Liu, Hong Fan

**Affiliations:** ^1^Department of Respiratory Medicine and Critical Care Medicine, West China Hospital/West China School of Medicine, Sichuan University, Chengdu, China; ^2^The Center of Gerontology and Geriatrics, West China Hospital/West China School of Medicine, Sichuan University, Chengdu, Sichuan, China

**Keywords:** community-acquired pneumonia, fluoroquinolones, β-lactams, macrolides, systematic review, meta-analysis, randomized controlled trial

## Abstract

**Background:** The choice of empirical antibiotic treatment for patients with community-acquired pneumonia (CAP) who are admitted to non-intensive care unit (ICU) hospital wards is complicated by the limited availability of evidence. We systematically reviewed the efficacy and safety of strategies of empirical treatment with respiratory fluoroquinolone monotherapy and β-lactam with or without macrolide for non-ICU hospitalized CAP patients.

**Methods:** We searched databases including PubMed, the Cochrane Library (Issue11, 2018), EMbase, China National Knowledge Internet (CNKI), WanFang Data, VIP, and China Biology Medicine disc (CBMdisc) to identify randomized controlled trials (RCTs) involving the comparison of respiratory fluoroquinolone monotherapy and β-lactam with or without macrolide for the non-ICU hospitalized patients with CAP up to November 2018. Two reviewers independently screened literature according to the inclusion and exclusion criteria, extracted data, and assessed the risk of bias of the included studies. A meta-analysis was performed with the outcomes.

**Results:** A total of 22 studies involving 6,235 patients were included. The results of the meta-analysis showed a non-significant trend toward an advantage to the respiratory fluoroquinolone in overall mortality (RR 0.82, 95% CI 0.65–1.02). No significant difference was found between the two strategies in clinical success (the intention-to-treat population: RR 1.03, 95% CI 0.99–1.08; the clinically evaluable population: RR 1.03, 95% CI 0.999–1.055; the population in which it was unclear whether intention-to-treat or per-protocol analysis was used: RR 1.04, 95% CI 0.99–1.09), microbiological treatment success (RR 1.04, 95% CI 0.997–1.092), and length of stay (SMD −0.06, 95% CI −0.16 to 0.04). The advantage of respiratory fluoroquinolone was statistically significant on the drug-related adverse events (RR 0.87, 95% CI 0.77–0.97).

**Conclusions:** Current evidence shows that fluoroquinolone monotherapy has similar efficacy and favorable safety compared with β-lactam with or without macrolide for non-ICU hospitalized CAP patients. Since the limitation of region, quantity and quality of included studies, more RCTs with large scale and high quality are needed to verify the above conclusion.

## Introduction

Long recognized as a major cause of death, community-acquired pneumonia (CAP) has been studied intensively since the late 1800s (Musher and Thorner, 2014). Despite the development of antimicrobial agents, pneumonia remains a major cause of hospitalization and death worldwide (Thomas et al., 2012; Welte et al., 2012).

Physicians must choose an optimal therapeutic regimen that eliminates the infection effectively, minimizes the risk of developing drug resistance and does not compromise the safety of the patient. Guidelines were written to develop a uniform set of recommendations that would provide appropriate antimicrobial therapy for the majority of patients with CAP. For patients with CAP who are admitted to a non-intensive-care-unit (ICU) ward, most guidelines recommend either respiratory fluoroquinolone monotherapy or β-lactam with or without macrolide for empirical treatment (Mandell et al., 2007; Lim et al., 2009; Woodhead et al., 2011; Cao et al., 2018). In America, guidelines recommend a respiratory fluoroquinolone monotherapy or a β-lactam plus a macrolide for the non-ICU inpatients (Mandell et al., 2007). In Britain, the British Thoracic Society suggests that amoxicillin is preferred for adults hospitalized with low severity CAP, while amoxicillin plus a macrolide is preferred for patients hospitalized with moderate severity CAP (levofloxacin, moxifloxacin, or doxycyline is alternative agent for those intolerant of penicillins or macrolides) (Lim et al., 2009). In Europe, guidelines recommend a respiratory fluoroquinolone monotherapy (levofloxacin or moxifloxacin), or a non-antipseudomonal cephalosporin, or a β-lactam (e.g., aminopenicillin) with or without a macrolide for non-ICU hospitalized patients (Woodhead et al., 2011). In China, a β-lactam (e.g., penicillins-β-lactamase-inhibitor combinations) with or without a macrolide, or respiratory fluoroquinolone monotherapy is suggested for the non-ICU inpatients (Cao et al., 2018). However, there is no consensus on which strategy is the best one. Level-one evidence for the comprehensive comparison of the two strategies is limited.

As main classes of antibiotics that have dominated the market for years, β-lactams, macrolides and fluoroquinolones are active against the major causative agents of CAP with different mechanisms (Walsh, 2003; Raja et al., 2004; Suda et al., 2018). β-lactam antibiotics work by inhibiting cell wall biosynthesis (inhibiting the β-lactam “binding protein” enzymes) in the bacterial organism (Fisher et al., 2005). They are effective against major causative bacteria of CAP (e.g., Streptococcus pneumonia) but not effective against Mycoplasma Pneumoniae (MP) or Chlamydia Pneumoniae (CP). Macrolides inhibit protein biosynthesis by binding to the P site on the 50S subunit of the bacterial ribosome and they are effective against Legionella Pneumophila, mycoplasma and chlamydia (Tenson et al., 2003). Physicians usually prescribe β-lactam plus macrolide for patients with CAP when infection with MP or CP is suspected. Fluoroquinolones eradicate bacteria by inhibiting the replication and transcription of bacterial DNA (preventing bacterial DNA from unwinding and duplicating) (Hooper, 2001; Aldred et al., 2014). Fluoroquinolones, especially respiratory fluoroquinolones (moxifloxacin, gemifloxacin, and levofloxacin) act against the major causative agents of CAP (including major causative bacteria, MP, CP and Legionella Pneumophila) and they are widely used as a monotherapy for patients with CAP.

Researchers from different countries and areas have performed randomized controlled trials (RCTs) to compare the efficacy of the two strategies. However, the results were not consistent. Finch et al. found that monotherapy with moxifloxacin was superior to that with a standard combination regimen of a β-lactam with or without a macrolide in the treatment of patients with CAP admitted to a hospital (Finch et al., 2002). Similarly, Huang G et al. reported that moxifloxacin was superior to cefuroxime with azithromycin in inpatients with low-moderate severity CAP (Huang et al., 2008). On the contrary, Erard et al. found that there were no significant differences between levofloxacin monotherapy and ceftriaxone with or without clarithromycin in non-ICU hospitalized CAP patients (Erard et al., 2004). Li BH et al. also reported that no significant differences were found between levofloxacin and cefuroxime with azithromycin in non-ICU hospitalized CAP patients (Li et al., 2009). Additionally, the small amount of patients enrolled in each trial limited the validity of the results.

Therefore, we conducted a systematic review and meta-analysis to conclusively and comprehensively compare the efficacy and safety of respiratory fluoroquinolone monotherapy vs. β-lactam with or without macrolide for empirical treatment for non-ICU hospitalized CAP patients.

## Methods

### Search Strategy

We searched databases including PubMed, the Cochrane Library (Issue11, 2018), EMbase, CNKI, WanFang Data, VIP and China Biology Medicine disc (CBMdisc) to identify RCTs up to November 2018. Search terms were “community-acquired pneumonia,” “fluoroquinolones” or “levofloxacin” or “moxifloxacin” or “gemifloxacin,” and “macrolides” or “β-lactams.” The search was restricted to RCTs. The language of the research papers was restricted to English and Chinese. All reference lists from relevant articles and reviews were hand-searched for additional eligible studies. We did not include abstracts from conferences because there is frequently considerable difference between data presented in conference abstracts and the subsequent peer-reviewed publications.

### Study Selection

Two reviewers (SL and XT) independently carried out the literature search and examined relevant RCTs for further assessment. A checklist was used to assess whether studies met our inclusion criteria: (1) population: hospitalized patients diagnosed with CAP; (2) exposure: one of levofloxacin, moxifloxacin or gemifloxacin; (3) comparison group: β-lactams with or without macrolides; (4) outcome: at least include one of mortality, clinical treatment success, microbiological treatment success, length of hospital stay or adverse events; (5) study design: RCTs. Exclusion criteria eliminated duplicate reports and studies on patients aged < 18 years, outpatients, critically ill patients admitted to ICU, or patients identified as having some form of healthcare-associated pneumonia (HCAP).

### Data Extraction

Two reviewers (SL and XT) independently extracted data from the trials included in the meta-analysis using a predesigned review form. In case of any disagreement between the two reviewers, a third reviewer extracted the data and the results were attained by consensus. The authors of trials were contacted for missing data when necessary. Data on first author, publication details, study design, included population, drug tested, endpoint data and adverse events during the treatment were extracted.

### Assessment of Risk of Bias

Two reviewers (SL and XT) independently assess the risk of bias of the RCTs included in the meta-analysis. We use the domain-based method as recommended in The Cochrane Hand-book (Higgins and Altman, 2011a) according to: sequence generation, allocation concealment, blinding, incomplete outcome data addressed, free of selective reporting, and free of other bias. A third review author was responsible for resolving disagreements.

### Outcomes

The primary outcome was all-cause mortality during the study period (treatment and follow-up period). Secondary outcomes included: clinical treatment success (“cure” was defined as resolution of all symptoms and signs of infections; “improvement” was defined as resolution of two or more of the baseline symptoms or signs of infections) (Frank et al., 2002; Writing Group of Guidance for Clinical Trials of Anti-bacterial Drugs, 2014) assessed at the test-of-cure (TOC) visit in the intention-to-treat population and clinically evaluable population; microbiological treatment success (defined as the eradication of baseline pathogens, or as presumed eradication based on the clinical outcomes when post-treatment cultures were not performed) (Frank et al., 2002; Writing Group of Guidance for Clinical Trials of Anti-bacterial Drugs, 2014); length of hospital stay; and adverse events probably related to the study regimens. Data was extracted preferentially by intention to treat.

### Data Analysis and Statistical Methods

Heterogeneity was examined using the χ^2^ test (*P* ≤ 0.1) and the I^2^ test (I^2^ > 50% defining significant inconsistency). Publication bias was assessed using the funnel plot method and Egger's test. Risk ratios (RRs) were calculated for individual trials, with 95% confidence intervals (CIs). Meta-analysis was conducted using the Mantel–Haenszel fixed-effects model. We compared the fixed-effect model to a random-effects model when we observed significant heterogeneity between the trials (*P* ≤ 0.10). The results from the fixed-effects model are presented only when there was no significant heterogeneity between trials (*P* > 0.1); otherwise, the results from the random-effects model are presented. Analyses were conducted using Stata 11.0. For studies with multiple treatment groups, we assessed intervention groups for relevance for our review. If more than two groups were relevant, we combined groups to create a single pair-wise comparison.

## Results

### Study Selection Process

**Figure 1:**
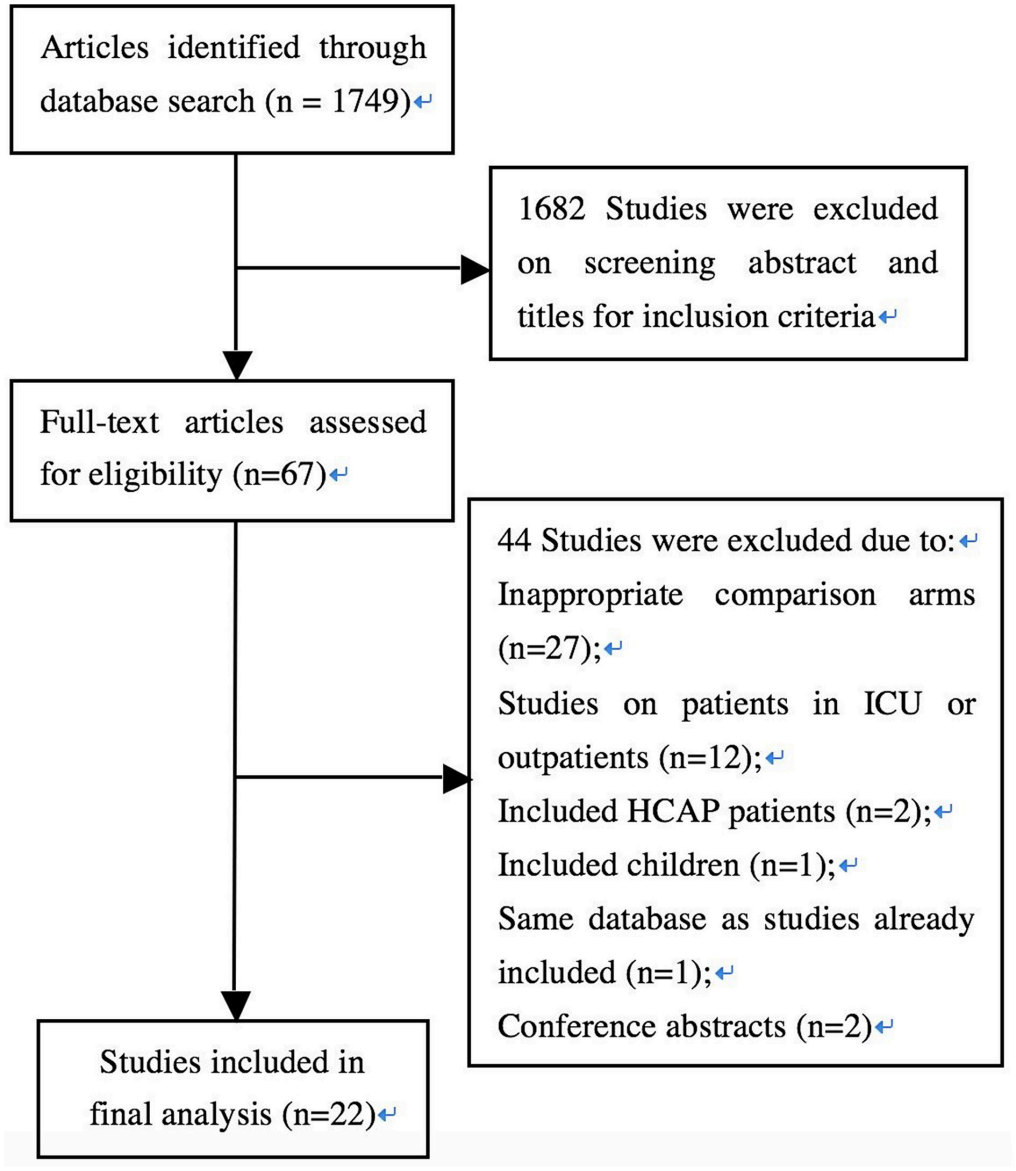
Flow diagram of the selection of studies for inclusion in the meta-analysis.

The flow diagram in Figure 1 shows the detailed screening and selection process applied before including trials in the meta-analysis. We identified a total of 1,749 citations from biomedical databases. After screening all titles and/or abstracts, 67 studies were identified for full text review. Forty-four studies were subsequently excluded for the following reasons: inappropriate comparison arms (*n* = 27); studies on patients in ICU or outpatients (*n* = 12); including HCAP patients (*n* = 2); including children (*n* = 1); same database as studies already included (*n* = 1); conference abstracts (*n* = 2). Twenty-two full-text publications involving 6,235 patients were ultimately identified (Finch et al., 2002; Frank et al., 2002; Lode et al., 2002; Erard et al., 2004; Leophonte et al., 2004; Zervos et al., 2004; Portier et al., 2005; Welte et al., 2005; Chang et al., 2006; Xu et al., 2006; Zhang et al., 2006; Lin et al., 2007; Zhao and Chen, 2007; Huang et al., 2008; Shao et al., 2008; Gao et al., 2009; Li et al., 2009; Yang and Zhang, 2009; Han et al., 2010; Lee et al., 2012; Liu et al., 2012; Postma et al., 2015).

### Study Characteristics

The main characteristics of the included trials are shown in Table 1. The trials were carried out between 1997 and 2013 in more than 25 countries. With a mean or median age between 47 and 77 years, the patients enrolled were mainly Caucasian and Asian and mostly from European counties, China, and the United States (US). Data on the comparison of respiratory fluoroquinolone monotherapy with β-lactam monotherapy was available in two trials (Leophonte et al., 2004; Postma et al., 2015), β-lactam–macrolide combination therapy in 16 trials (Frank et al., 2002; Zervos et al., 2004; Portier et al., 2005; Xu et al., 2006; Zhang et al., 2006; Lin et al., 2007; Zhao and Chen, 2007; Huang et al., 2008; Shao et al., 2008; Gao et al., 2009; Li et al., 2009; Yang and Zhang, 2009; Han et al., 2010; Lee et al., 2012; Liu et al., 2012), and β-lactam with or without macrolide (β-lactam ± macrolide) in five trials (Finch et al., 2002; Lode et al., 2002; Erard et al., 2004; Welte et al., 2005; Chang et al., 2006). Patients received sequential intravenous to oral or intravenous antibiotics in 20 trials (Finch et al., 2002; Frank et al., 2002; Lode et al., 2002; Erard et al., 2004; Zervos et al., 2004; Welte et al., 2005; Chang et al., 2006; Xu et al., 2006; Zhang et al., 2006; Lin et al., 2007; Zhao and Chen, 2007; Shao et al., 2008; Gao et al., 2009; Li et al., 2009; Yang and Zhang, 2009; Han et al., 2010; Lee et al., 2012; Liu et al., 2012; Postma et al., 2015). Treatment was given orally initially in two trials (Leophonte et al., 2004; Portier et al., 2005). We did not find publication bias in the performed analyses.

**Table 1:** Study characteristics.

**Study**	**Location**	**Population**	**Enrolled patients**	**Age (years)**	**Drug tested**		**Duration (d)**	**Funding source**
			**FQ/β±M**	**FQ/β±M**	**FQ**	**β±M**		
Chang et al., 2006	China	Asian	41/41	47 (18–70)	Sequential i.v. levofloxacin 400 mg OD followed by p.o. levofloxacin 100 mg t.i.d.	Sequential i.v. cefuroxime 1,500 mg b.i.d. followed by p.o. cefuroxime axetil 500 mg b.i.d. ± p.o. roxithromycin 150 mg b.i.d.	7–10	NS
Erard et al., 2004	Switzerland	Caucasian	79/37	77 (24–92)/77 (26–95)	p.o. levofloxacin 500 mg q12h	Sequential i.v. and p.o. ceftriaxone 2 g OD ± i.v./p.o. clarithromycin 500 mg q12h	7–10	Aventis
Finch et al., 2002	Belgium, France, Germany, Greece, Israel, South Africa, Spain, Switzerland, Russia, UK	Mixed	301/321	55.2 ± 20.6/55.9 ± 19.6	Sequential i.v. and p.o. moxifloxacin 400 mg OD	Sequential i.v. 1.2 g and p.o. 625 mg co-amoxiclav t.i.d. ± i.v./p.o. clarithromycin 500 mg b.i.d.	7–14	NS
Frank et al., 2002	USA	Mixed	115/121	67.8 ± 13.11/67.3 ± 13.17	i.v./p.o. levofloxacin 500 mg OD	i.v. ceftriaxone 1 g OD + i.v. azithromycin 500 mg OD	≥10	NS
Gao et al., 2009	China	Asian	40/38	55.2 ± 12.3/54.3 ± 13.6	Sequential i.v. and p.o. moxifloxacin 400 mg OD	i.v. cefuroxime 2g b.i.d. + p.o. azithromycin 500 mg OD	7–14	NS
Han et al., 2010	China	Asian	40/40	47.95 ± 15.13/47.85 ± 15.85	i.v. moxifloxacin 400 mg OD	i.v. ceftriaxone 3 g OD + i.v. azithromycin 500 mg OD	7–10	NS
Huang et al., 2008	China	Asian	119/65	71.4 ± 5.0/72.7 ± 5.4	i.v. moxifloxacin 400 mg OD or Sequential i.v. and p.o. moxifloxacin 400 mg OD	i.v. cefuroxime 3 g b.i.d. + i.v. azithromycin 500 mg OD	7–10	NS
Lee et al., 2012	Korea	Asian	20/20	54 ± 20/53 ± 16	Sequential i.v. and p.o. levofloxacin 750 mg OD	i.v. ceftriaxone 2 g OD + p.o. azithromycin 500 mg OD, followed by p.o. cefpodoxime 200 mg/D	NS	Daiichi-Sankyo Korea
Leophonte et al., 2004	France, Poland, South Africa	Mixed	167/153	53.3 ± 20.4/55.3 ± 19.8	p.o. gemifloxacin 320 mg OD	p.o. amoxicillin/clavulanate 1 g/125 mg t.i.d.	7–10	NS
Li et al., 2009	China	Asian	40/35	55.1 ± 12.5/54.2 ± 13.1	i.v. levofloxacin 500 mg OD	i.v. cefuroxime 2g b.i.d. + azithromycin 500 mg OD	7–14	NS
Lin et al., 2007	Taiwan	Asian	26/24	65.3 ± 13.2/71.0 ± 11.4	Sequential i.v. and p.o. levofloxacin 500 mg OD	Sequential i.v. 500 mg/100 mg and p.o. 250 mg/125 mg amoxicillin/clavulanate q8h + p.o. azithromycin 500 mg q12h	7–14	Daiichi
Liu et al., 2012	China	Asian	33/33	73 ± 11.48/72 ± 8.78	i.v. moxifloxacin 400 mg OD	i.v. cefoperazone/sulbactam 2.5 g b.i.d. + i.v. azithromycin 0.5 g OD	7–14	NS
Lode et al., 2002	US, Poland, Canada, Germany, Italy, UK, Australia, Austria, Belgium, Guatemala, Hungary, Lebanon, Philippines, Singapore, Switzerland	Mixed	172/173	59.5 ± 17.7/58.2 ± 18.7	p.o. gemifloxacin 320 mg OD	Sequential i.v. ceftriaxone 2 g OD followed by p.o. cefuroxime 500 mg b.i.d. ± macrolide	7–14	GSK
Portier et al., 2005	France	Caucasian	174/175	59.3 ± 17.9/62.4 ± 18.0	p.o. moxifloxacin 400 mg OD	p.o. amoxicillin-clavulanate 1,000/125 mg t.i.d. + p.o. roxithromycin 150 mg b.i.d.	10	Bayer
Postma et al., 2015	the Netherlands	Caucasian	888/1395	71 ± 14.81/70 ± 14.87	moxifloxacin or levofloxacin	Beta-lactam (amoxicillin, amoxicillin plus clavulanate, or a third-generation cephalosporin) monotherapy and combined with macrolide (azithromycin, erythromycin, or clarithromycin)	NS	NS
Shao et al., 2008	China	Asian	199/199	47.43 ± 18.94/51.50 ± 19.95	Sequential i.v. and p.o. levofloxacin 500 mg OD	Sequential i.v. cefuroxime 1500 mg b.i.d. followed by p.o. cefuroxime axetil 500 mg b.i.d. + p.o. azithromycin 500 mg t.i.d.	10–14	NS
Welte et al., 2005	Germany, France, Greece, Lithuania, and Poland	Caucasian	200/197	NS	Sequential i.v. and p.o. moxifloxacin 400 mg OD	i.v. ceftriaxone 2 g OD ± i.v. erythromycin 1 g q6-8h	7–14	Bayer Vital GmbH
Xu et al., 2006	China	Asian	20/20	NS	i.v. moxifloxacin 400mg OD	i.v. cefoperazone 2 g b.i.d. + i.v. azithromycin 0.5 g OD	7–14	NS
Yang and Zhang, 2009	China	Asian	50/50	72.9/73.3	i.v. moxifloxacin 400 mg OD	i.v. ceftriaxone 2 g OD + i.v. azithromycin 0.5 g OD	7	NS
Zervos et al., 2004	US, Canada, and Europe	Mixed	112/107	72.8 ± 13.6/70.7 ± 13.5	i.v. levofloxacin 500 mg OD	i.v. ceftriaxone 1 g OD + i.v. azithromycin 500 mg OD	7–14	Pfizer and Pliva
Zhang et al., 2006	China	Asian	50/50	58.1 ± 11.7/56.8 ± 12.4	i.v. levofloxacin 300 mg b.i.d.	i.v. ceftriaxone 1 g b.i.d. + p.o. azithromycin 500 mg OD	7–14	NS
Zhao and Chen, 2007	China	Asian	30/25	55.2 ± 12.3/54.3 ± 13.6	i.v. levofloxacin 500 mg OD	i.v. cefuroxime 2.25 g b.i.d. + p.o. azithromycin 500 mg OD	7–14	NS

*FQ, fluoroquinolone; β±M, β-lactam with or without macrolide; NS, not specified; i.v., intravenous; p.o., oral; OD, once daily; b.i.d., twice daily; t.i.d., three times daily*.

Sequence generation (specified rule for allocating interventions to participants based on some random process) (Higgins and Altman, 2011a) was adequate in 6 studies (Frank et al., 2002; Welte et al., 2005; Lin et al., 2007; Li et al., 2009; Lee et al., 2012; Postma et al., 2015) and no information was available for other studies. With numbered sachets, only Léophonte's study (Leophonte et al., 2004) reported adequate allocation concealment (steps taken to secure strict implementation of random assignments by preventing foreknowledge of the forthcoming allocations) (Higgins and Altman, 2011a). Insufficient information was available for the other studies. One trial (Leophonte et al., 2004) was double-blinded and the remaining were open label. Details of the incomplete data for each outcome will be discussed in the following sections. We did not find any specific concerns over selective reporting. For other potential source of bias, we found that seven studies (Lode et al., 2002; Erard et al., 2004; Zervos et al., 2004; Portier et al., 2005; Welte et al., 2005; Lin et al., 2007; Lee et al., 2012) were sponsored by pharmaceutical companies, which might generate bias in the assessment of outcomes. Besides, one study (Postma et al., 2015) was a cluster-randomized, crossover trial comparing treatment strategies assigned to hospitals in defined study periods as the unit of randomization. Analyses in this study took into account cluster-period effects and center effects.

### Mortality

**Figure 2:**
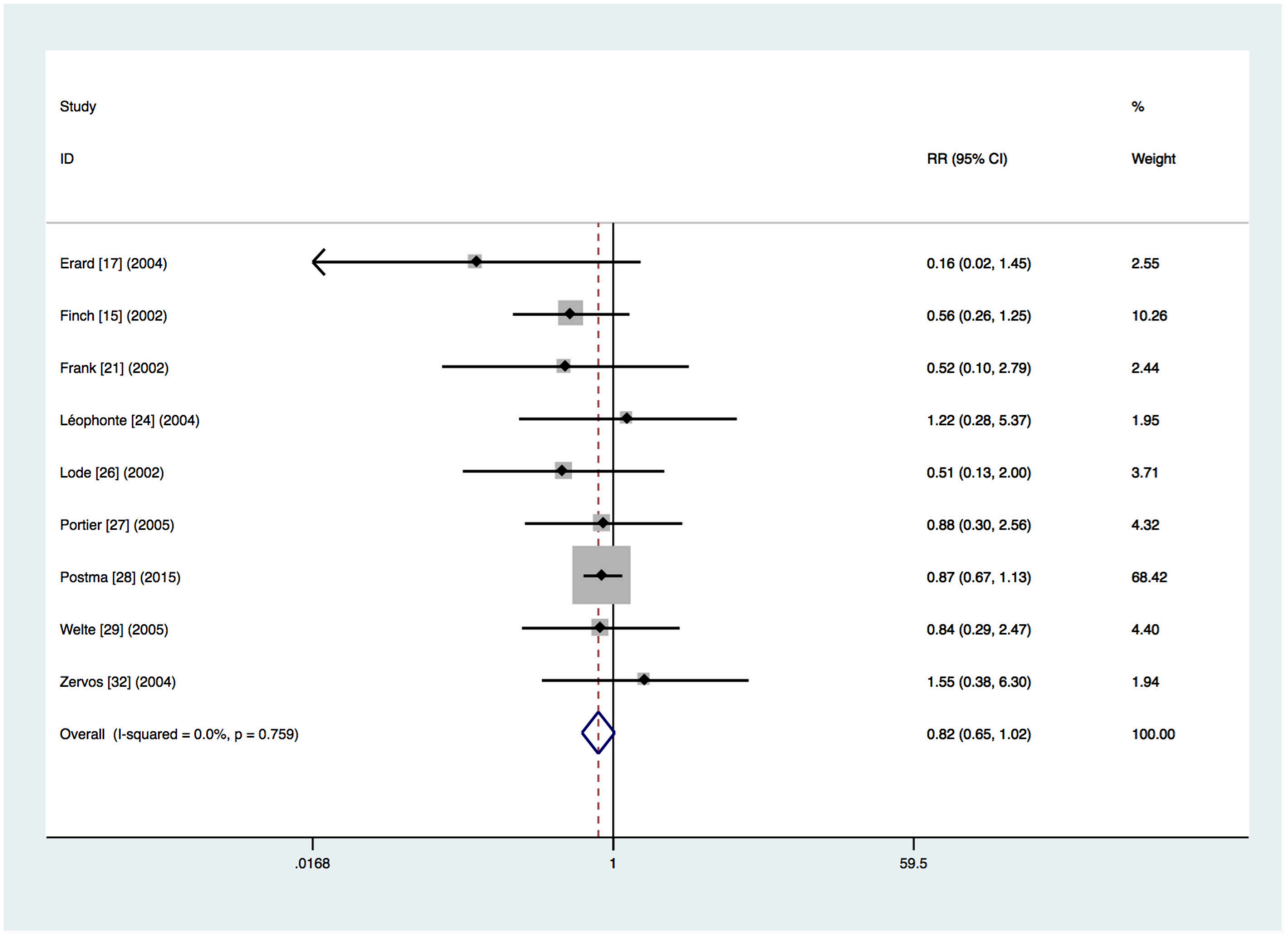
Mortality for respiratory fluoroquinolone monotherapy vs. β-lactam with or without macrolide. A fixed-effect Mantel–Haenszel (M–H) meta-analysis is shown with results presented as risk ratios with 95% confidence intervals (CIs).

Nine trials provided mortality outcomes (Finch et al., 2002; Frank et al., 2002; Lode et al., 2002; Erard et al., 2004; Leophonte et al., 2004; Zervos et al., 2004; Portier et al., 2005; Welte et al., 2005; Postma et al., 2015). In total, 114 (5.2%) of the 2,198 patients in the respiratory fluoroquinolone group and 191 (7.2%) of the 2,670 patients in the comparator group died during the course of the studies. A non-significant trend toward an advantage to the respiratory fluoroquinolone group was observed (RR 0.82, 95% CI 0.65–1.02) (Figure 2). No heterogeneity was observed (*I*^2^ = 0%).

Data about mortality of patients with β-lactam monotherapy was available for 2 trials (Leophonte et al., 2004; Postma et al., 2015) and no significant difference was found (RR 0.99, 95% CI 0.72–1.35). The non-significant advantage of the respiratory fluoroquinolone group was seen in the patients with β-lactam–macrolide combination therapy from 4 trials (RR 0.81, 95% CI 0.62–1.06) (Frank et al., 2002; Zervos et al., 2004; Portier et al., 2005; Postma et al., 2015). However, mortality rate was significantly lower in the respiratory fluoroquinolone group among patients with β-lactam ± macrolide regimen from 4 trials (RR 0.56, 95% CI 0.33–0.98) (Finch et al., 2002; Lode et al., 2002; Erard et al., 2004; Welte et al., 2005).

The same non-significant advantage of the respiratory fluoroquinolone group was seen when we excluded the cluster-randomized cross-over trial (RR = 0.70, 95% CI 0.46–1.07) (Postma et al., 2015).

### Clinical Treatment Success

**Figure 3:**
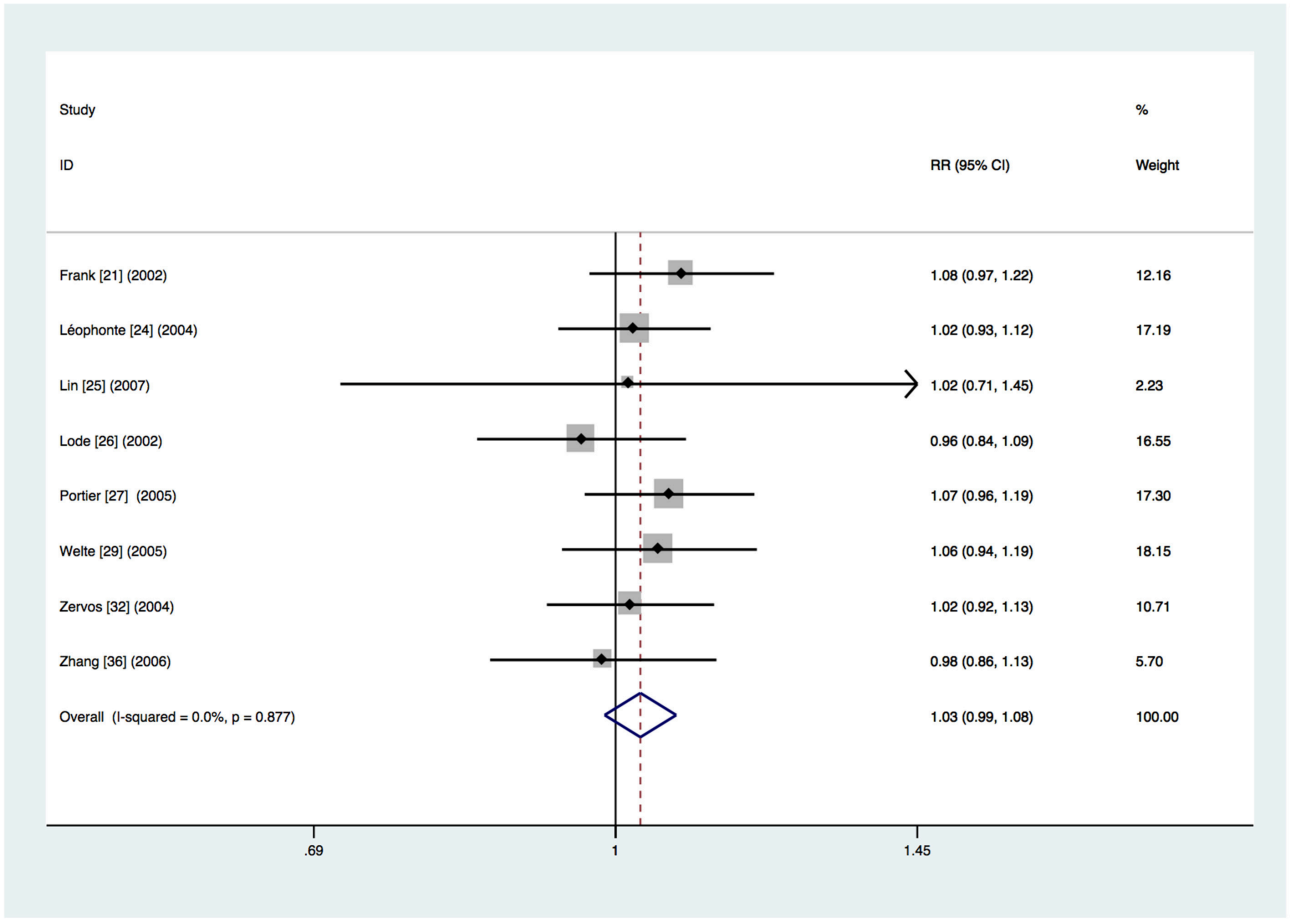
Clinical treatment success analysis based on intention-to-treat population.

Data about clinical treatment success in the intention-to-treat population were available for 8 trials (Frank et al., 2002; Lode et al., 2002; Leophonte et al., 2004; Zervos et al., 2004; Portier et al., 2005; Welte et al., 2005; Zhang et al., 2006; Lin et al., 2007). Overall, treatment with respiratory fluoroquinolone was successful for 804 (80.9%) of the 994 patients. Treatment with comparator antibiotics was successful for 775 (78.4%) of the 988 patients. Meta-analysis showed that there was no significant difference (RR 1.03, 95% CI 0.99–1.08) (Figure 3). No heterogeneity was observed (*I*^2^ = 0%). The same conclusion was drawn from separate analyses of the studies on β-lactam–macrolide combination therapy (RR = 1.05, 95% CI 0.99–1.11) (Frank et al., 2002; Zervos et al., 2004; Portier et al., 2005; Zhang et al., 2006; Lin et al., 2007) and β-lactam ± macrolide regimen (RR 1.01, 95% CI 0.92–1.10) (Lode et al., 2002; Welte et al., 2005). Only one study (Leophonte et al., 2004) used β-lactam monotherapy and thus a combined analysis could not be performed. No significant difference was found in studies where treatment was given orally (RR 1.05, 95% CI 0.98–1.12) (Leophonte et al., 2004; Portier et al., 2005) or initially intravenously (RR 1.02, 95% CI 0.97–1.08) (Frank et al., 2002; Lode et al., 2002; Zervos et al., 2004; Welte et al., 2005; Zhang et al., 2006; Lin et al., 2007). No significant difference was found in the trials funded by pharmaceutical companies (RR 1.03, 95% CI 0.97–1.09) (Lode et al., 2002; Zervos et al., 2004; Portier et al., 2005; Welte et al., 2005; Lin et al., 2007) or not (RR 1.04, 95% CI 0.97–1.11) (Frank et al., 2002; Leophonte et al., 2004; Zhang et al., 2006).

**Figure 4:**
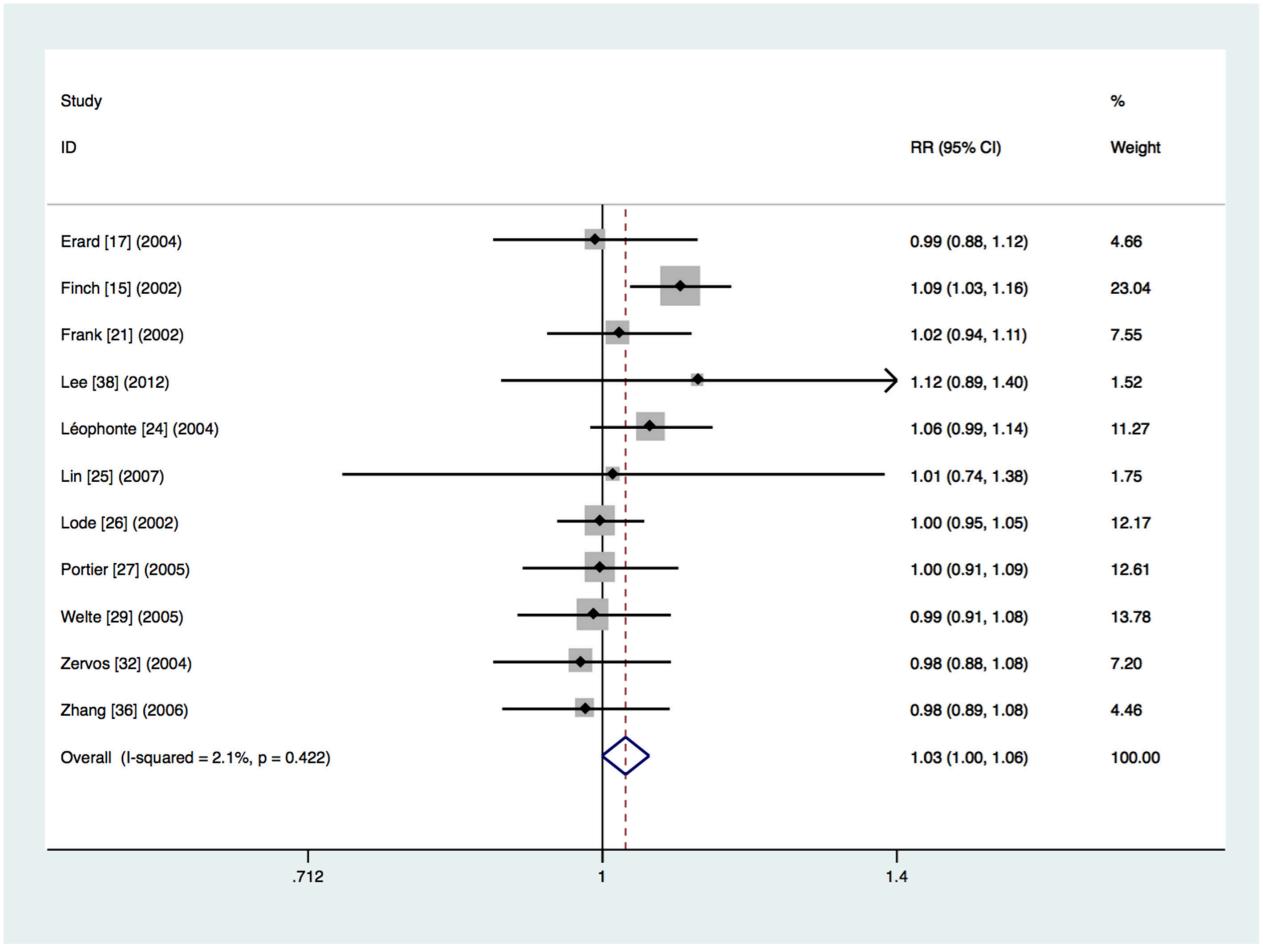
Clinical treatment success analysis based on clinically evaluable population.

Eleven trials provided data about clinical treatment success in the clinically evaluable population (Finch et al., 2002; Frank et al., 2002; Lode et al., 2002; Erard et al., 2004; Leophonte et al., 2004; Zervos et al., 2004; Portier et al., 2005; Welte et al., 2005; Zhang et al., 2006; Lin et al., 2007; Lee et al., 2012). The clinical treatment success was 91.3% (1,048 of the 1,148 patients) in the respiratory fluoroquinolone group and 88.9% (984 of the 1,107 patients) in the comparator antibiotics group. Meta-analysis showed that there was no significant difference (RR 1.03, 95% CI 0.999–1.055) (Figure 4). No significant heterogeneity was observed (*I*^2^ = 2.1%). The same conclusion was drawn from separate analyses of the studies on β-lactam–macrolide combination therapy (RR 1.00, 95% CI 0.96–1.05) (Frank et al., 2002; Zervos et al., 2004; Portier et al., 2005; Zhang et al., 2006; Lin et al., 2007; Lee et al., 2012) and β-lactam ± macrolide regimen (RR 1.02, 95% CI 0.97–1.08) (Finch et al., 2002; Lode et al., 2002; Erard et al., 2004; Welte et al., 2005). Only one study used β-lactam monotherapy (Leophonte et al., 2004). No significant difference was found in studies where treatment was given orally (RR 1.03, 95% CI 0.97–1.09) (Leophonte et al., 2004; Portier et al., 2005) or initially intravenously (RR 1.03, 95% CI 0.996–1.059) (Finch et al., 2002; Frank et al., 2002; Lode et al., 2002; Erard et al., 2004; Zervos et al., 2004; Welte et al., 2005; Zhang et al., 2006; Lin et al., 2007; Lee et al., 2012). No significant difference was found in the trials funded by pharmaceutical companies (RR 1.00, 95% CI 0.96–1.04) (Lode et al., 2002; Erard et al., 2004; Zervos et al., 2004; Portier et al., 2005; Welte et al., 2005; Lin et al., 2007; Lee et al., 2012). However, the advantage of respiratory fluoroquinolone was statistically significant in the studies not funded by pharmaceutical companies (RR 1.06, 95% CI 1.02–1.10) (Finch et al., 2002; Frank et al., 2002; Leophonte et al., 2004; Zhang et al., 2006).

**Figure 5:**
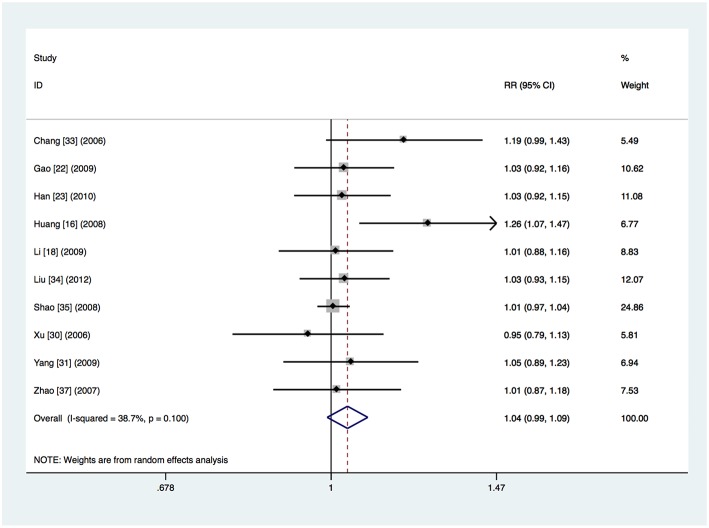
Clinical treatment success analysis for the studies in which it was unclear whether intention-to-treat or per-protocol analysis was used.

It was unclear whether intention-to-treat or per-protocol analysis was used in ten studies, which did not refer to dropouts or reported the total number of dropouts but did not give the numbers per study arm (Chang et al., 2006; Xu et al., 2006; Zhao and Chen, 2007; Huang et al., 2008; Shao et al., 2008; Gao et al., 2009; Li et al., 2009; Yang and Zhang, 2009; Han et al., 2010; Liu et al., 2012). The clinical treatment success was 93.7% (565 of the 603 patients) in the respiratory fluoroquinolone group and 89.5% (479 of the 535 patients) in the comparator antibiotics group. Heterogeneity was detected (*I*^2^ = 38.7%, *P* = 0.10) and meta-analysis done by the random-effects model showed no significant difference (RR 1.04, 95% CI 0.99–1.09) (Figure 5). The advantage of respiratory fluoroquinolone was statistically significant when compared with β-lactam–macrolide combination therapy (RR 1.05, 95% CI 1.01–1.09) (Xu et al., 2006; Zhao and Chen, 2007; Huang et al., 2008; Shao et al., 2008; Gao et al., 2009; Li et al., 2009; Yang and Zhang, 2009; Han et al., 2010; Liu et al., 2012) and the heterogeneity was reduced in this analysis (*I*^2^ = 25.8%, *P* = 0.21). Only one study used β-lactam ± macrolide regimen (Chang et al., 2006) and no trials used β-lactam monotherapy. Treatment was given initially intravenously in all trials. Not any study was funded by pharmaceutical companies.

### Microbiological Treatment Success

Eighteen studies reported microbiological treatment success outcomes (Finch et al., 2002; Frank et al., 2002; Lode et al., 2002; Leophonte et al., 2004; Zervos et al., 2004; Portier et al., 2005; Chang et al., 2006; Xu et al., 2006; Zhang et al., 2006; Lin et al., 2007; Zhao and Chen, 2007; Huang et al., 2008; Shao et al., 2008; Gao et al., 2009; Li et al., 2009; Yang and Zhang, 2009; Han et al., 2010; Lee et al., 2012). In the total microbiologically evaluable population, 513 (88.8%) of the 578 patients/isolates in the respiratory fluoroquinolone group and 462 (85.2%) of the 542 patients/isolates in the comparator group achieved eradication or presumed eradication of the baseline pathogens. The most common pathogens were *S. pneumoniae, H. influenza*, and *M. pneumoniae*. Details about drug resistance were reported in 9 trials (Finch et al., 2002; Frank et al., 2002; Lode et al., 2002; Erard et al., 2004; Leophonte et al., 2004; Zervos et al., 2004; Portier et al., 2005; Zhang et al., 2006; Postma et al., 2015). For respiratory fluoroquinolone, only one *S. aureus* isolate resistant to levofloxacin was found. *S. pneumoniae* strains resistant to the comparator antibiotics were more commonly found. Resistance was more prominent among macrolides than among β-lactams.

There was no significant difference in the overall microbiological treatment success rates between the two groups (RR 1.04, 95% CI 0.997–1.092). No significant heterogeneity was observed (*I*^2^ = 10.3%). The same conclusion was drawn from separate analyses of the studies on β-lactam–macrolide combination therapy (RR 1.05, 95% CI 0.98–1.12) (Frank et al., 2002; Zervos et al., 2004; Portier et al., 2005; Xu et al., 2006; Zhang et al., 2006; Lin et al., 2007; Zhao and Chen, 2007; Huang et al., 2008; Shao et al., 2008; Gao et al., 2009; Li et al., 2009; Yang and Zhang, 2009; Han et al., 2010; Lee et al., 2012) and β-lactam ± macrolide regimen (RR 1.07, 95% CI 0.99–1.15) (Finch et al., 2002; Lode et al., 2002; Chang et al., 2006). Only one study used β-lactam monotherapy (Leophonte et al., 2004). No significant difference was found in studies where treatment was given orally (RR 0.98, 95% CI 0.88–1.10) (Leophonte et al., 2004; Portier et al., 2005). In studies where treatment was given initially intravenously, the advantage of respiratory fluoroquinolone was statistically significant (RR 1.05, 95% CI 1.003–1.108) (Finch et al., 2002; Frank et al., 2002; Lode et al., 2002; Zervos et al., 2004; Chang et al., 2006; Xu et al., 2006; Zhang et al., 2006; Lin et al., 2007; Zhao and Chen, 2007; Huang et al., 2008; Shao et al., 2008; Gao et al., 2009; Li et al., 2009; Yang and Zhang, 2009; Han et al., 2010; Lee et al., 2012).

In addition, there was no significant difference between the respiratory fluoroquinolone group and the comparator group for the microbiological treatment success rates of *S. pneumoniae* (343 isolates, RR 0.99, 95% CI 0.85–1.17) (Finch et al., 2002; Frank et al., 2002; Lode et al., 2002; Leophonte et al., 2004; Zervos et al., 2004; Portier et al., 2005; Xu et al., 2006; Zhang et al., 2006; Lin et al., 2007; Han et al., 2010; Lee et al., 2012), *H. influenzae* (113 isolates, RR 1.04, 95% CI 0.87–1.25) (Finch et al., 2002; Frank et al., 2002; Lode et al., 2002; Leophonte et al., 2004; Zervos et al., 2004; Xu et al., 2006; Zhang et al., 2006; Lin et al., 2007; Han et al., 2010), *M. pneumoniae* (77 isolates, RR 1.08, 95% CI 0.96–1.23) (Finch et al., 2002; Lode et al., 2002; Han et al., 2010; Lee et al., 2012), *C. pneumoniae* (41 isolates, RR 1.03, 95% CI 0.83–1.27) (Finch et al., 2002; Lode et al., 2002; Han et al., 2010) and *Legionella* species (21 isolates, RR 0.99, 95% CI 0.60–1.63) (Finch et al., 2002; Lode et al., 2002; Leophonte et al., 2004).

### Length of Hospital Stay

Data about the length of stay in hospital were available in 9 trials (Finch et al., 2002; Lode et al., 2002; Erard et al., 2004; Zervos et al., 2004; Welte et al., 2005; Lin et al., 2007; Shao et al., 2008; Li et al., 2009; Postma et al., 2015). Four trials provided the median duration of hospital stay and 0–2 days less duration was found in the respiratory fluoroquinolone group (Lode et al., 2002; Erard et al., 2004; Welte et al., 2005; Postma et al., 2015). Six trials provided the mean duration of hospital stay and no significant difference was found (SMD −0.06, 95% CI −0.22 to 0.11) (Finch et al., 2002; Zervos et al., 2004; Welte et al., 2005; Lin et al., 2007; Shao et al., 2008; Li et al., 2009). Among these studies, one trial provided both the median and the mean duration (Welte et al., 2005). Using the statistic methods recommended in the Cochrane Hand-book (Higgins and Altman, 2011b), we calculated the mean duration for all trials and performed an overall meta-analysis. No significant difference was found (SMD −0.06, 95% CI −0.16 to 0.04). Heterogeneity was moderate (*I*^2^ = 45.6%). However, the advantage of respiratory fluoroquinolone was statistically significant when compared with β-lactam ± macrolide regimen (SMD −0.18, 95% CI −0.28 to −0.07) (Finch et al., 2002; Lode et al., 2002; Erard et al., 2004; Welte et al., 2005) and the heterogeneity was reduced in this analysis (*I*^2^ = 9.7%). No significant difference was found when respiratory fluoroquinolone was compared with β-lactam–macrolide combination therapy (SMD 0.03, 95% CI −0.06 to 0.11) (Zervos et al., 2004; Lin et al., 2007; Shao et al., 2008; Li et al., 2009; Postma et al., 2015). Data of patients with β-lactam monotherapy was only available in one trial (Postma et al., 2015).

### Adverse Events

All but two trials reported on drug-related adverse outcomes. One trial did not refer to adverse events (Lin et al., 2007). One trial reported on complications while data on drug-related adverse outcomes was unavailable (Postma et al., 2015). The majority of the adverse events were mild to moderate. The most commonly studied adverse effects were gastrointestinal events (including nausea, diarrhea and vomiting) and liver function abnormalities. However, the definition of gastrointestinal events differed, some including all the three symptoms (nausea, diarrhea and vomiting) and some nausea alone, thereby excluding an accurate comparison for each symptom alone. QTc prolongation was reported in one trial with one patient in the co-amoxiclav ± clarithromycin group.

The advantage of respiratory fluoroquinolone was statistically significant on the adverse events (RR 0.87, 95% CI 0.77–0.97). No significant heterogeneity was observed (*I*^2^ = 25.9%). The same conclusion was drawn from analysis of the studies on serious adverse events (RR 0.67, 95% CI 0.51–0.88) (Finch et al., 2002; Frank et al., 2002; Leophonte et al., 2004; Zervos et al., 2004; Portier et al., 2005; Welte et al., 2005). The percentage of patients who were withdrawn from the trials because of adverse events was not significantly different between the two groups (RR 0.87, 95% CI 0.59–1.30) (Finch et al., 2002; Frank et al., 2002; Lode et al., 2002; Erard et al., 2004; Zervos et al., 2004; Welte et al., 2005; Chang et al., 2006; Liu et al., 2012). Respiratory fluoroquinolone was associated with significantly fewer adverse events compared with β-lactam–macrolide combination therapy (RR 0.74, 95% CI 0.61–0.90) (Frank et al., 2002; Zervos et al., 2004; Portier et al., 2005; Xu et al., 2006; Zhang et al., 2006; Zhao and Chen, 2007; Huang et al., 2008; Shao et al., 2008; Gao et al., 2009; Li et al., 2009; Yang and Zhang, 2009; Han et al., 2010; Lee et al., 2012; Liu et al., 2012). No significant difference was found when respiratory fluoroquinolone was compared with β-lactam ± macrolide regimen (RR 0.99, 95% CI 0.74–1.34) (Finch et al., 2002; Lode et al., 2002; Erard et al., 2004; Welte et al., 2005; Chang et al., 2006). Only one study used β-lactam monotherapy (Leophonte et al., 2004).

Gastrointestinal events were reported in 16 studies and were significantly less common in the respiratory fluoroquinolone group (RR 0.63, 95% CI 0.43 to 0.94) (Finch et al., 2002; Frank et al., 2002; Lode et al., 2002; Erard et al., 2004; Leophonte et al., 2004; Portier et al., 2005; Welte et al., 2005; Chang et al., 2006; Xu et al., 2006; Zhang et al., 2006; Huang et al., 2008; Shao et al., 2008; Gao et al., 2009; Han et al., 2010; Lee et al., 2012; Liu et al., 2012). Non-significant advantage of respiratory fluoroquinolone was found with regard to liver function abnormalities (RR 0.73, 95% CI 0.52 to 1.03) (Finch et al., 2002; Lode et al., 2002; Leophonte et al., 2004; Welte et al., 2005; Zhang et al., 2006; Zhao and Chen, 2007; Shao et al., 2008; Gao et al., 2009; Li et al., 2009; Yang and Zhang, 2009; Lee et al., 2012).

## Discussion

This systematic review with meta-analysis compared the efficacy and safety of respiratory fluoroquinolone monotherapy and β-lactam with or without macrolide for non-ICU hospitalized CAP patients. A non-significant trend toward an advantage to respiratory fluoroquinolone was observed on overall mortality. No significant difference was found between the two strategies in clinical success, microbiological treatment success, and length of stay. The advantage of respiratory fluoroquinolone was statistically significant in the drug-related adverse events. The advantage of respiratory fluoroquinolone in clinical treatment success was statistically significant in the studies not funded by pharmaceutical companies based on the clinically evaluable population (RR 1.06, 95% CI 1.02–1.10) and the advantage in microbiological treatment success was statistically significant in the studies where treatment was given initially intravenously (RR 1.05, 95% CI 1.003–1.108). The results were consistent with those of the primary analysis for the subgroup of β-lactam–macrolide combination therapy except for the clinical success based on the data that it was unclear whether intention-to-treat or per-protocol analysis was used (RR 1.05, 95% CI 1.01–1.09). Analysis was available only in mortality for the subgroup of β-lactam monotherapy and no significant difference was found. For the subgroup of β-lactam ± macrolide regimen, respiratory fluoroquinolone was associated with significantly lower mortality and less length of stay, while no significant difference was found in clinical treatment success, microbiological treatment success and adverse events.

An earlier meta-analysis performed by Vardakas et al. (2008) investigated whether respiratory quinolone monotherapy was superior to other recommended antimicrobial regimens, including combination therapy consisting of a macrolide and β-lactam as well as monotherapy (macrolide, ketolide, or β-lactam alone), for the treatment of adults with CAP. While no significant difference was found in mortality, clinical success rates were significantly higher and adverse events were significantly fewer with fluoroquinolone monotherapy. However, we found no significant difference in the overall clinical treatment success. In our meta-analysis, we focused on direct comparison of respiratory fluoroquinolone monotherapy and β-lactam with or without macrolide for non-ICU hospitalized CAP patients, precluding the interference from outpatients or patients in ICU and the interference from other drugs. Furthermore, we included new trials performed in recent years, providing greater statistical confidence for our meta-analysis.

The moderate total mortality rates in the two groups of our meta-analysis (5.2% and 7.2%) supports the opinion that the patients admitted to non-ICU hospital wards are associate with moderate risk of death (Mandell et al., 2007; Lim et al., 2009). A non-significant trend toward an advantage to the respiratory fluoroquinolone group was observed and more RCTs are needed to further verify the result.

Overall, no significant difference was found in clinical treatment success. The advantage of respiratory fluoroquinolone was statistically significant in some subgroup analyses. However, we noticed that the advantage was not obvious (RR = 1.06 and RR = 1.05). Therefore, we considered that the advantages of respiratory fluoroquinolone in these subgroup analyses were limited in clinical significance.

Drug resistance was found more prominent in the comparator antibiotics and most commonly among macrolides, which was in correspondence with previous surveillance (Mandell et al., 2007; Ho et al., 2009; Lim et al., 2009). There was no significant difference in the microbiological treatment success. However, the amount of patients enrolled in the analysis was limited (578/542 patients/isolates) and the patients included in the analysis for atypical pathogens were mainly from European countries. Previous surveillance results showed that the resistance of *M. pneumoniae* to macrolides in Asian countries was significantly higher than in the European or North American countries (Mandell et al., 2007; Lim et al., 2009; Mikasa et al., 2016; Cao et al., 2018). Since the drug resistance pattern differs greatly in different areas and countries, more RCTs with large scale in different areas are needed to verify the above conclusion.

When respiratory fluoroquinolone was compared with β-lactam–macrolide combination therapy, no significant difference was found in mortality, clinical treatment success, microbiological treatment success and length of stay. Respiratory fluoroquinolone was associated with fewer adverse events. When respiratory fluoroquinolone was compared with β-lactam monotherapy, no significant difference was found in mortality. Because of the lack of studies in this subgroup, analyses for other outcomes were not available. This may be because researchers used β-lactam monotherapy mainly in outpatients with low severity and usually added macrolides for hospitalized patients with moderate to severe pneumonia. More studies or detailed data comparing respiratory fluoroquinolone with β-lactam monotherapy in hospitalized CAP patients under supervision are needed. In the studies with β-lactam ± macrolide regimen as control group, respiratory fluoroquinolone was associated with significantly lower mortality rate and less length of stay. No significant difference was found in clinical treatment success, microbiological treatment success and adverse events. As the comparator regimens in these studies were not exactly the same, the results of this subgroup analysis might introduce more bias and thus provided relatively less statistical confidence.

There were several limitations in our meta-analysis. First, our findings may be affected by the quality of trials included in the analysis. Sequence generation was adequate in 6 studies. Only one trial was double-blinded, and one trial reported adequate allocation concealment. A sensitivity analysis was performed including only trials that reported adequate sequence generation. The results were consistent with those of the primary analysis except for overall adverse events rate, which indicated non-significant advantage of the respiratory fluoroquinolone group. Second, the quantity of studies included in some subgroup analyses was small, resulting in limited statistical confidence. Third, we failed to perform a comprehensive analysis for β-lactam monotherapy because of the lack of studies comparing respiratory fluoroquinolone with it. Finally, seven studies were sponsored by pharmaceutical companies, which might generate bias in the assessment of outcomes. Sensitivity analyses limited to industry-funded and not industry-funded studies were performed. The results showed that for the clinical treatment success in the clinically evaluable population, the advantage of respiratory fluoroquinolone was statistically significant in the studies not funded by pharmaceutical companies but limited in clinical significance. For the overall adverse events, no significant difference was found in the studies not funded by pharmaceutical companies. Other analyses indicated similar findings with the primary analyses.

In conclusion, despite the limitations of our meta-analysis, we conclude that respiratory fluoroquinolone monotherapy has similar efficacy and favorable safety compared with β-lactam with or without macrolide for non-ICU hospitalized CAP patients. Since the limitation of region, quantity and quality of included studies, more RCTs with large scale and high quality are needed to verify the above conclusion.

## Author Contributions

HF, SL, and XT conceived and designed the studies. SL and XT carried out the literature search, extracted data, assess the risk of bias of the RCTs. YM, DW, JH, LZ, MW, LW, and TL helped conduct the analyses. SL wrote the manuscript. All authors reviewed the manuscript.

### Conflict of Interest Statement

The authors declare that the research was conducted in the absence of any commercial or financial relationships that could be construed as a potential conflict of interest.
